# Comparative outcomes of PreserFlo^TM^MicroShunt in primary open-angle and pseudoexfoliative glaucoma: A matched analysis

**DOI:** 10.1371/journal.pone.0357501

**Published:** 2026-09-02

**Authors:** Leonie Bourauel, Benjamin Aretz, Michael Petrak, Frank G. Holz, Karl Mercieca, Constance Liegl

**Affiliations:** 1 Department of Ophthalmology, University of Bonn, Bonn, Germany; 2 Institute for Medical Biometry, Informatics and Epidemiology, University of Bonn, Bonn, Germany; 3 Department of Ophthalmology, Klinikum Dortmund, University Witten/Herdecke, Dortmund, Germany; Federal Medical Centre, Asaba, NIGERIA

## Abstract

**Purpose:**

Comparing outcomes of PreserFlo^TM^ MicroShunt in eyes with primary open-angle (POAG) and pseudoexfoliative glaucoma (PEXG) through a matched analysis.

**Methods:**

Retrospective matched-pair analysis of patients undergoing PreserFlo^TM^ MicroShunt (PFMS) due to POAG (n = 25) and PEXG (n = 25). Success rates (A/B: IOP ≤ 21 / 18 mmHg + IOP reduction ≥20% from baseline; C: IOP ≤ 15 mmHg + IOP reduction ≥25%; D: IOP ≤ 12 mmHg + IOP reduction ≥30%) and secondary outcomes were evaluated.

**Results:**

Fifty eyes of 49 patients were included. At 12 months, complete and qualified success rates for Criteria A – D were 68.0% and 96.0%, 68.0% and 96.0%, 64.0% and 72.0%, and 32.0% and 32.0%, respectively, in the POAG group, compared with 44.0% and 76.0%, 40.0% and 72.0%, 32.0% and 52.0%, and 8.0% and 12.0%, respectively, in the PEXG group (p = 0.039, 0.024, 0.189, and 0.271, respectively). Mean preoperative IOP decreased from 25.12 mmHg (POAG) and 25.64 mmHg (PEXG, p = 0.98) to 12.64 mmHg and 15.04 mmHg postoperatively (p = 0.043). The mean number of IOP-lowering drops was significantly lower in eyes with POAG (0.52 ± 1.01) than with PEXG (1.3 ± 1.55; p = 0.031) after 12 months. Eyes with PEXG had significantly higher rates of postoperative complications than eyes with POAG (p = 0.038) with no vision-threatening sequelae.

**Conclusions:**

PFMS is a safe and effective procedure for reducing IOP in eyes with POAG and PEXG, with lower IOP levels being achieved in eyes with POAG. Significantly more postoperative complications and interventions were needed in eyes with PEXG.

## Introduction

Glaucoma is a major cause of irreversible vision loss, impacting patients’ quality of life [1,2]. Treatment strategies depend on severity, with surgery recommended when intraocular pressure (IOP) lowering medications or laser therapy are insufficient [3,4]. Minimally invasive bleb forming surgeries have emerged as alternatives to conventional filtering surgery in selected patients because of their favourable postoperative outcomes and safety profiles [5–8].

The PreserFlo® MicroShunt (PFMS) (Santen, Miami, USA) is one such device, designed to drain aqueous humour into the subconjunctival space, forming a filtering bleb. PFMS has emerged as a treatment option for patients with glaucoma progression despite topical IOP-lowering medications, particularly in cases not requiring very low target IOPs [9]. Two and three-year studies indicate that PFMS is generally safe and effective, lowering IOP to the mid-to-low teens and maintaining this reduction over the long term [6,10–12]. However, research on PFMS outcomes in specific glaucoma subtypes remains limited, highlighting the need for further investigation.

Among the various forms of glaucoma, pseudoexfoliative glaucoma (PEXG) is of particular interest. The latter is characterized by deposition of fibrillary material in the anterior segment of the eye, especially on the anterior lens capsule and at the pupillary margin [13,14] and is associated with higher baseline IOP, more advanced visual field defects and a tendency for more rapid progression compared to primary open-angle glaucoma (POAG) [15,16]. From a pathophysiological perspective, PEXG eyes exhibit chronically elevated aqueous humour concentrations of pro-fibrotic mediators alongside a constitutively impaired blood-aqueous barrier [17,18]. This pro-inflammatory and pro-fibrotic milieu promotes postoperative Tenon’s fibroblast activation and bleb scarring, which may affect long-term filtration function and partly account for worse surgical outcomes observed in PEXG [17,19]. Furthermore, there is a risk that fibrillary material may also deposit within the lumen of the PFMS at its anterior chamber entry point, physically impeding aqueous outflow and contributing to differential outcomes between POAG and PEXG [20].

Despite its clinical significance, relatively few studies have specifically examined PFMS outcomes in PEXG in particular. A comparative study suggests that PFMS is non-inferior to trabeculectomy in PEXG patients [21]. Additionally, similar efficacy and safety profiles of PFMS have been reported in PEXG and POAG [20,22]. However, a matched comparative analysis between the two glaucoma subtypes has not yet been performed. This study therefore aims to compare PFMS outcomes in a matched real-world cohort of POAG and PEXG patients.

## Materials and methods

### Patients

The study protocol conformed to the ethical guidelines of the 2000 Declaration of Helsinki as reflected in a priori approval by the Ethics Committee of the Medical Faculty, Rheinische Friedrich-Wilhelms-Universität Bonn, Germany (reference number 458/21). As this was a retrospective study, the requirement for informed consent was waived by the Ethics Committee.

The medical records of all patients who underwent PFMS surgery for POAG or PEXG at the Department of Ophthalmology, University Hospital of Bonn, Germany, between April 2021 and January 2024 were reviewed. Patient data were assessed for research purposes between February 3^rd^ and 14^th^ 2025. Patients were included in the study if they were 18 years or older, had a diagnosis of POAG or PEXG, had a PFMS implanted in at least one eye and had at least 12 months of follow-up data. As only 25 patients with a PEXG met the requirements, they were pair-matched at baseline with successive POAG cases, thus each eye with PEXG was matched to an eye with POAG from our database with regards to preoperative IOP, number of glaucoma medication, age and gender. A history of prior glaucoma surgery was not an exclusion criterion in order to represent a real-world cohort. After the 25 pairs had been formed, all patients were pseudonymized so that identification was no longer possible. Preoperative data documented for each patient included gender, age, glaucoma type, BCVA, clinical features such as IOP, glaucoma medication, gonioscopy and anterior segment findings, while follow-up data included BCVA, IOP, visual fields, complications, and postoperative glaucoma medication details. Each included patient underwent a comprehensive ophthalmic examination at presentation, which included best-corrected visual acuity (BCVA) assessment using a Snellen chart (converted to logMAR for statistical analysis), IOP measurement via Goldmann applanation tonometry, slit lamp biomicroscopy, fundus biomicroscopy, and visual field testing using the Humphrey 24−2 visual field test strategy (Carl Zeiss Meditec, Inc., Dublin, CA).

### Surgical technique

All patients gave informed consent prior to surgery. Two experienced glaucoma surgeons followed a standardized surgical technique based on local protocol and previous descriptions [23]. All procedures were performed under sub-Tenon’s or subconjunctival anaesthesia. A traction suture in the superior quadrant was used to fix the cornea and a fornix-based conjunctival flap was created. Bleeding vessels were minimally cauterized before applying Mitomycin C (MMC) soaked corneal sponges under the sub-Tenon’s pocket. MMC at a concentration of 0.2 mg/ml was applied for two minutes. A 2 mm long scleral tunnel was then created 3 mm behind the marked limbus with a special 1 mm micrometre and the anterior chamber was entered with a 25-gauge needle. The implant was inserted through the tunnel with the fins positioned in the sclera. The Tenon’s and conjunctival layers were pushed over the implant, and both layers were reattached in their anatomical position with 10–0 nylon sutures. Postoperatively, patients were prescribed a standardized regimen of topical antibiotics and steroids, which were tapered over a 2–3 month period.

### Success criteria

We followed the recommendation of the ‘Guidelines on Design and Report of Glaucoma Surgical Trials’ of the World Glaucoma Association in defining four different success criteria [24]. These were based on different IOP thresholds: 1) Criterion-A: IOP ≤ 21 mmHg, reduction ≥20%; 2) Criterion-B: IOP ≤ 18 mmHg, reduction ≥20%; 3) Criterion C: IOP ≤ 15 mmHg, reduction ≥30%; 4) Criterion-D: IOP ≤ 12 mmHg, reduction ≥30%. Success was defined as complete if reached without glaucoma medication and as qualified if reached with glaucoma medication.

Failure of PFMS was considered when the above-mentioned criteria were not fulfilled at any post-operative visit after three months or if one of the following occurred: hypotony-related complications; IOP below 6 mmHg or over 21 mmHg; inadequate IOP control requiring acetazolamide; further glaucoma surgery or loss of light perception. A revision of the PFMS was not rated failure. Further, if a patient was revised, the IOP measured after revision was used to evaluate whether the patient met the respective success criterion. The WGA recommends scoring as failure if criteria are not met on two consecutive visits. As only 12-month data can be reported here, we have opted for a stricter definition.

Primary outcome included success rates based on the criteria above. Secondary outcomes were IOP, BCVA, number of IOP-lowering medications, complications and revision procedures. All analyses were conducted on a de-identified data set. Additionally, we used the same success criteria as in the studies previously conducted by our group and other studies on PFMS in order to facilitate comparison of the outcomes.

### Statistical analysis

Statistical analysis was performed with GraphPad Prism 10.4.1 (GraphPad Software, Boston, MA, USA) and R 4.4.2 [25]. BCVA values were converted to the logMAR scale prior to statistical analysis. Time-dependent survival probabilities were estimated with the Kaplan-Meier method and the log-rank test was used to compare subgroups. Survival times were calculated and reported in 95% confidence intervals (CIs). The Fisher’s exact test was used to compare the distributions of the nominal- or ordinal-scaled variables. The t-test was used for normal distributions and Mann-Whitney U-test was used for non-normal distributions in order to compare independent groups.

To account for the repeated, longitudinal nature of IOP measurements and to identify independent predictors of postoperative IOP control, a multivariable generalized additive mixed model (GAM) was fitted. The dependent (outcome) variable was postoperative IOP (mmHg), log-transformed to satisfy model assumptions, measured repeatedly at day 1 and months 1, 3, 6, and 12 after surgery. A Gaussian error distribution with an identity link function was specified for the log-transformed outcome. The following fixed-effect covariates were included in the model: glaucoma diagnosis (PEXG vs. POAG), sex, age category (≤75, 76–83, ≥ 84 years), number of preoperative antiglaucoma medications, previous SLT treatment, previous glaucoma surgery, preoperative BCVA (logMAR-based: normal <0.3, mild 0.3 - 0.5, moderate 0.6 - 1.0, severe 1.1 - 1.3, (near) blindness ≥2.0), and postoperative follow-up time point (day 1, month 1, 3, 6, 12). Preoperative IOP was modeled as a smooth (penalized spline) term to allow for a potentially nonlinear relationship with postoperative IOP, consistent with the generalized additive component of the model. To account for within-patient correlation from repeated measurements, a patient-specific random intercept was included as the random-effects component of the model. Variance inflation factors (VIFs) were calculated for all fixed-effect predictors and indicated no relevant multicollinearity. Effect estimates from the GAM are reported as regression coefficients (β) with corresponding 95% confidence intervals. All statistical tests were two-sided, and p-values <0.05 were considered statistically significant.

## Results

We conducted a matched-pair analysis comparing eyes with PEXG and POAG, including 25 eyes from 24 patients with PEXG and 25 eyes from 25 patients with POAG. There were no significant differences in terms of age, sex, ethnicity, type of anaesthesia or pre-existing conditions. Similarly, the prevalence of prior glaucoma surgeries was comparable (6 eyes, 24% for POAG vs. 7 eyes, 28% for PEXG). Further details and patient characteristics are shown in Table 1.

**Table 1:** Demographics and clinical characteristics of patients with POAG and PEXG undergoing PFMS surgery.

Comparison of POAG and PEXG patients with PFMS			
	POAG	PEXG	P value
	25 eyes, 25 pat. n (%)	25 eyes, 24 pat. n (%)	
**Gender**			1.00
Female / Male	16 / 9 (64 / 36)	16 / 9 (64 / 36)	
**Ethnicity**			1.00
Caucasian	25 (100)	25 (100)	
**Age**			0.14
Mean (SD)	79.44 (8.33)	82.64 (6.5)	
**Blood thinning therapy**			0.77
Yes	8 (32.0)	10 (28.0)	
**Arterial Hypertension**			0.11
Yes	15 (60.0)	21 (84.0)	
**Diabetes mellitus Type II**			0.99
Yes	6 (24.0)	5 (20.0)	
**Anaesthesia**			0.99
Local	22 (88.0)	21 (84.0)	
General	3 (12.0)	4 (16.0)	
**Previous glaucoma surgery**			0.99
Yes	6 (24.0)	7 (28.0)	
**Which surgery**			
Canaloplasty ab externo	1 (4.0)	1 (4.0)	
iStent	0 (0)	1 (4.0)	
Hydrus with concurrent Cataract surgery	1 (4)	0 (0)	
Trabectome	2 (8.0)	3 (12.0)	
MP-CPC	1 (4.0)	1 (4.0)	
XEN	0 (0)	1 (4.0)	
Trabeculectomy	1 (4.0)	0 (0)	
**Previous SLT**			1.00
Yes	2 (8.0)	2 (8.0)	
**Time since diagnosis (years)**			**0.02**
Mean (SD)	13.13 (7.66)	6.12 (4.12)	
**Previous vitrectomy**			1.00
Yes	0 (0)	0 (0)	
**Glaucoma drops**			1.00
Yes	25 (100.0)	25 (100)	
**Number of glaucoma drops**			0.63
Mean (SD)	3.4 (0.65)	3.36 (0.57)	
**Acetazolamide**			0.38
Yes	11 (44.0)	7 (28.0)	
**Lens status**			0.67
Phakic / Pseudophakic	2 (8.0); 23 (92.0)	4 (16.0) / 21 (84.0)	
**BCVA preoperatively (logMAR)**			0.49
Mean (SD)	0.54 (0.59)	0.66 (0.69)	
**IOP preoperatively**			0.98
Mean (SD)	25.12 (7.54)	25.64 (8.56)	
**Mean Deviation (MD) (Humphrey 24−2)**			0.82
Mean (SD)	−11.94 (10.14)	−12.71 (8.57)	
**Pattern Standard Deviation (PSD)**			0.89
Mean (SD)	7.25 (4.66)	7.46 (3.58)	
Visual Field Index			0.44
Mean (SD)	73.62 (27.42)	65.14 (28.51)	

**Fig 1:**
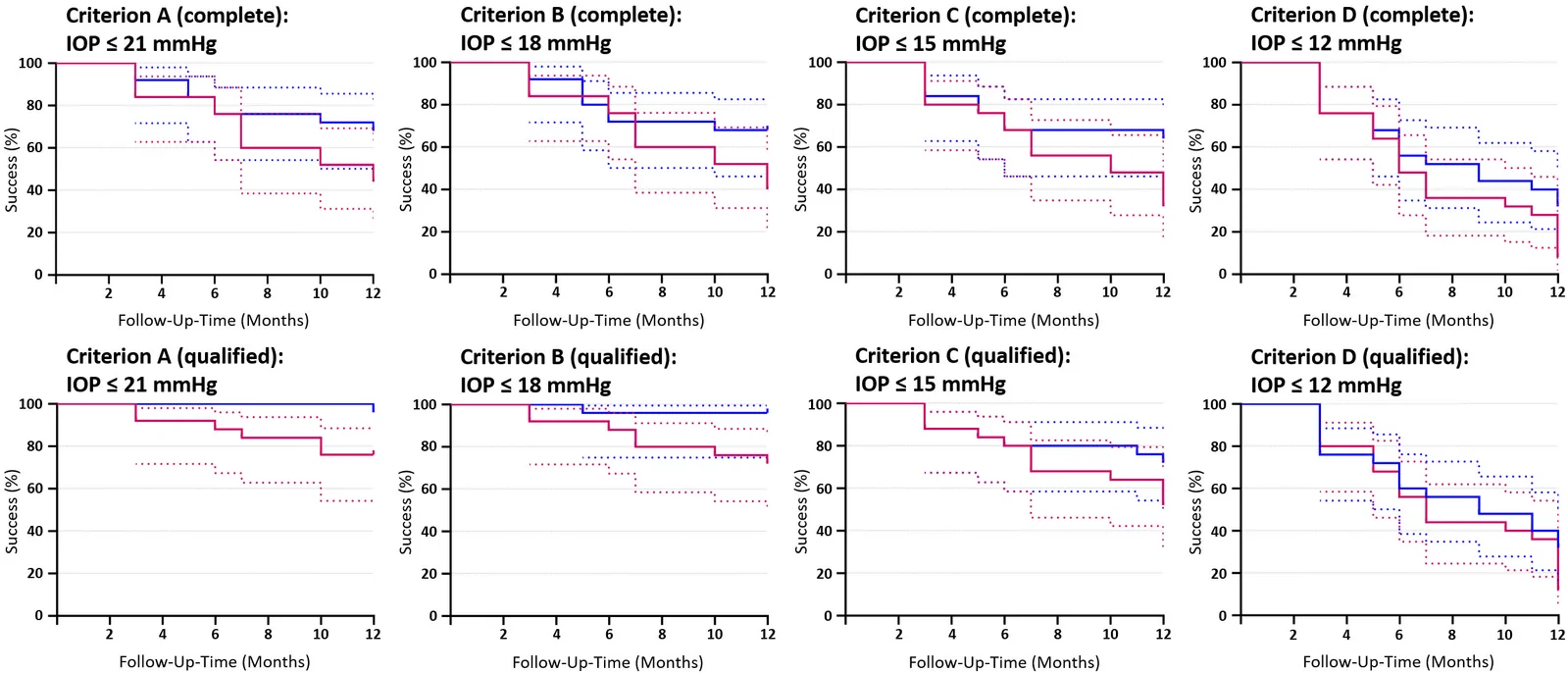
Kaplan Meier curves with success rates (95% CI) over 12 months postoperatively for POAG (blue line) and PEXG (dark red line).

Regarding the primary outcomes, significant differences were observed in qualified success rates for Criterion A (p = 0.039) and Criterion B (p = 0.024), while differences in the remaining criteria did not reach statistical significance. At 12 months, complete and qualified success rates were numerically higher in the POAG group than in the PEXG group across all four criteria (Fig 1). For Criterion A (IOP ≤ 21 mmHg), complete and qualified success rates were 68.0% (52.0 - 84.0) and 96.0% (88.0 - 100.0), respectively, in POAG compared with 44.0% (24.0 - 64.0) and 76.0% (56.0 - 92.0), respectively, in PEXG. For Criterion B (IOP ≤ 18 mmHg), rates were 68.0% (52.0 - 84.0) and 96.0% (88.0 - 100.0) in POAG compared with 40.0% (20.0 - 60.0) and 72.0% (52.0 - 88.0) in PEXG. For Criterion C (IOP ≤ 15 mmHg), complete and qualified success rates were 64.0% (48.0 - 84.0) and 72.0% (56.0 - 88.0) in POAG compared with 32.0% (16.0 - 52.0) and 52.0% (32.0 - 72.0) in PEXG. For Criterion D (IOP ≤ 12 mmHg), complete and qualified success rates were both 32.0% (16.0 - 51.9) in POAG compared with 8.0% (0.0 - 20.0) and 12.0% (0.0 - 24.0) in PEXG.

**Fig 2:**
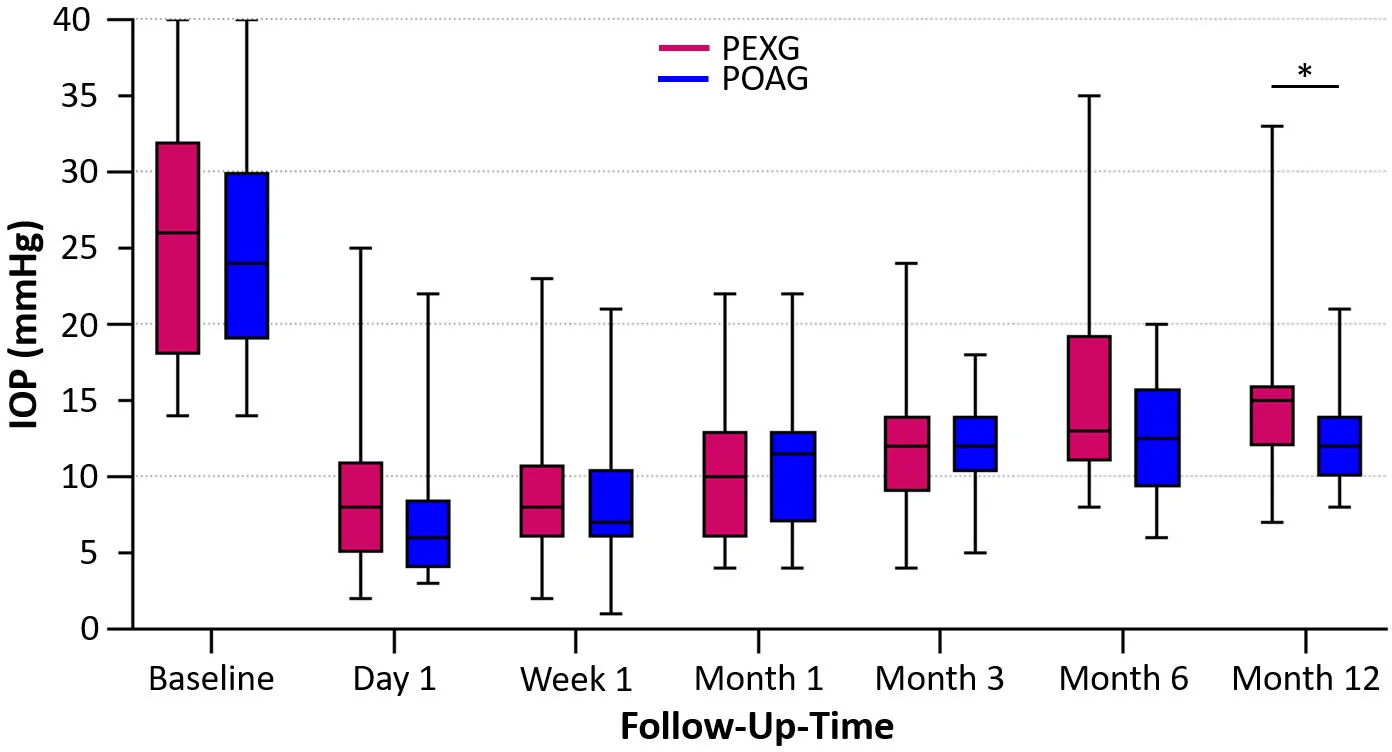
Development of the IOP over 12 months after PFMS surgery (mean ± SD, range).

Mean preoperative IOP was similar between the groups: 25.64 ± 8.56 mmHg in the PEXG group and 25.12 ± 7.54 mmHg in the POAG group (p = 0.98). Both groups showed substantial IOP reduction postoperatively. On day 1, mean IOP dropped to 7.32 ± 4.63 mmHg for POAG and 8.40 ± 5.23 mmHg for PEXG, representing percentage reductions of 71% and 66.8%, respectively (Table 2, Fig 2). By month 1, the IOP increased slightly to 10.58 ± 4.36 mmHg for POAG and 10.39 ± 4.7 mmHg for PEXG, with no significant difference. Although mean IOP remained higher in the PEXG group at 3 and 6 months, this difference was not statistically significant. At 12 months post-surgery, mean IOP was 12.64 ± 3.37 mmHg for POAG and 15.04 ± 5.13 mmHg for PEXG (p = 0.043), corresponding to percentage reductions of 49.8% and 40.5% respectively.

**Table 2:** Postoperative course of eyes with PFMS surgery.

*Follow-Up-Time*			
	POAG	PEXG	p
***Day 1 after surgery***			
**BCVA**			0.75
Mean (SD)	1.23 (0.8)	1.16 (0.84)	
**IOP**			0.35
Mean (SD)	7.32 (4.63)	8.4 (5.23)	
**Mean reduction of IOP (%)**	71.0	66.8	
Glaucoma drops			1.0
Yes (%)	0 (0)	0 (0)	
**Number of glaucoma drops**			1.0
Mean (SD)	0.0 (0)	0.0 (0)	
*Week 1 after surgery*			
**BCVA**			0.96
Mean (SD)	0.86 (0.72)	0.88 (0.74)	
**IOP**			0.57
Mean (SD)	7.92 (4.05)	8.71 (4.36)	
Mean reduction of IOP (%)	68.6	66.9	
**Glaucoma drops**			0.24
Yes (%)	0 (0)	2 (8.0)	
**Number of glaucoma drops**			0.24
Mean (SD)	0.00 (0)	0.13 (0.45)	
***Month 1 after surgery***			
**BCVA**			0.029
Mean (SD)	0.53 (0.51)	0.32 (0.36)	
**IOP**			0.68
Mean (SD)	10.58 (4.36)	10.39 (4.7)	
Mean reduction of IOP (%)	59.7	62.2	
**Glaucoma drops**			0.99
Yes (%)	1 (4.2)	1 (4.3)	
**Number of glaucoma drops**			0.99
Mean (SD)	0.08 (0.41)	0.04 (0.21)	
***Month 3 after surgery***			
**BCVA**			0.71
Mean (SD)	0.62 (0.70)	0.66 (0.66)	
**IOP**			0.69
Mean (SD)	11.92 (3.36)	12.48 (4.33)	
Mean reduction of IOP (%)	54.6	54.5	
Glaucoma drops			0.99
Yes (%)	2 (8.3)	2 (8.7)	
**Number of glaucoma drops**			0.99
Mean (SD)	0.13 (0.45)	0.13 (0.46)	
***Month 6 after surgery***			
**BCVA**			0.76
Mean (SD)	0.59 (0.70)	0.66 (0.72)	
**IOP**			0.16
Mean (SD)	12.46 (3.74)	15.32 (6.43)	
Mean reduction of IOP (%)	52.5	46.7	
***Glaucoma drops***			0.13
Yes (%)	6 (25.0)	11 (50.0)	
**Number of glaucoma drops**			0.091
Mean (SD)	0.5 (0.97)	1.09 (1.44)	
***Month 12 after surgery***			
**BCVA**			0.67
Mean (SD)	0.73 (0.74)	0.74 (0.78)	
**IOP**			**0.043**
Mean (SD)	12.64 (3.37)	15.04 (5.13)	
Mean reduction of IOP (%)	49.8	40.5	
**Glaucoma drops**			**0.038**
Yes (%)	5 (20.0)	12 (48.0)	
**Number of glaucoma drops**			**0.031**
Mean (SD)	0.52 (1.08)	1.30 (1.55)	
**Acetazolamide**			0.49
Yes (%)	0 (0)	1 (4.0)	

The mean number of IOP-lowering medications decreased in both groups from 3.40 ± 0.58 to 0.52 ± 1.01 in the POAG group and from 3.36 ± 0.57 agents to 1.30 ± 1.55 in the PEXG group after 12 months (p = 0.031). Preoperatively, 25 eyes (100%) in both groups received IOP-lowering medications and at 12 months postoperatively, five eyes (20.0%; POAG) compared to 12 eyes (48.0%; PEX) needed IOP-lowering medications (p = 0.038).

Postoperative complications occurred significantly more frequently in PEXG eyes (p = 0.038), though none resulted in vision threatening sequelae (Table 3). Most common complications for POAG were self-limiting hypotony with choroidal detachment in two eyes (8.0%) and vitreous haemorrhage in one eye (4.0%), with one case requiring viscoelastic injection into the anterior chamber (4.0%). In PEXG, complications included self-limiting hypotony (4.0%) and postoperative hyphema (8.0%). Among late complications (occurring after three months) contributing to surgical failure, bleb scarring was observed in one POAG eye (4.0%) and six PEXG eyes (24.0%), while corneal decompensation occurred in one PEXG eye (4.0%) only.

**Table 3:** Complications of PFMS surgery.

Time Point	Complications	POAG	PEXG
**Complications (overall; %) p = 0.038**		5 (20.0)	12 (48.0)
**<3 months postoperative**	Hypotony with shallow anterior chamber and injection of viscoelastic	1 (4.0)	–
	Self-limiting hypotony with choroidal detachment	2 (8.0)	1 (4.0)
	Hyphema	–	2 (8.0)
	Postoperative vitreous haemorrhage	1 (4.0)	–
	Corneal erosion	–	2 (8.0)
**>3 months postoperative**	Bleb scarring	1 (4.0)	6 (24.0)
	Corneal decompensation	–	1 (4.0)

Postoperative interventions were significantly more frequent in PEXG eyes (p = 0.023; Table 4). Four eyes (16.0%) with PEXG required a bleb revision, two (8.0%) underwent additional glaucoma procedures (PAUL glaucoma implant: one eye (4.0%), eyePlate implant: one eye (4.0%)) and two eyes (8.0%) required a bleb needling with 5-Fluoruracil. One eye (4.0%) in the POAG group underwent a bleb revision during the first postoperative year. Six eyes in the PEXG group and one eye in the POAG group were classified as failures due to an inadequately controlled IOP or a postoperative procedure (p = 0.09).

**Table 4:** Postoperative procedures.

Postoperative procedures	POAG	PEXG	P value
Postoperative procedures overall (%)	1 (4.0)	8 (32.0)	**0.023**
Revision (%) - thereof with EyePlate Implantation	1 (4.0) –	5 (20.0) 1 (4.0)	
Needling with 5-Fluoruracil	–	2 (8.0)	
PAUL glaucoma implant	–	1 (4.0)	

A GAM was used to evaluate factors associated with postoperative IOP over the 12-month follow-up period. Glaucoma type was significantly associated with postoperative IOP, with POAG eyes showing lower adjusted postoperative IOP than PEXG eyes (β = −0.230 [−0.349 to −0.110], p < 0.001). Among demographic variables, male sex (β = −0.141 [−0.238 to −0.044], p = 0.005) and older age were independently associated with lower adjusted postoperative IOP; within age categories, this association was significant for patients aged 76–83 years (vs. ≤ 75 years: β = −0.196 [−0.351 to −0.042], p = 0.013) and those aged ≥84 years (vs. ≤ 75 years: β = −0.214 [−0.354 to −0.073], p = 0.003). Among clinical variables, higher preoperative IOP was positively associated with postoperative IOP (β = 0.112 [0.099 to 0.125], p < 0.001), while a greater preoperative medication burden was associated with lower postoperative IOP (three agents vs. fewer than three: β = −0.439 [−0.672 to −0.206], p < 0.001; four agents vs. fewer than three: β = −0.362 [−0.606 to −0.117], p = 0.004). A history of prior glaucoma surgery was also associated with lower postoperative IOP (β = −0.139 [−0.248 to −0.031], p = 0.013). Regarding visual acuity, mild impairment (logMAR 0.3 - 0.5) and (near) blindness (logMAR ≥ 2.0) were each associated with lower postoperative IOP compared with normal vision (logMAR < 0.3; β = −0.198 [−0.305 to −0.090], p < 0.001 and β = −0.372 [−0.631 to −0.112], p = 0.005, respectively), whereas moderate impairment (logMAR 0.6 - 1.0: β = −0.057 [−0.165 to 0.279], p = 0.616) and severe impairment (logMAR 1.1 - 1.3: β = 0.095 [−0.144 to 0.334], p = 0.436) were not significantly associated with postoperative IOP. Previous SLT did not reach significance (β = −0.058 (−0.135 to 0.250), p = 0.560).

## Discussion

Various devices have been developed to lower IOP, providing alternatives to traditional glaucoma surgeries. Among these, PFMS received CE marking in 2012 and FDA approval in 2020. Studies demonstrated its safety and efficacy in reducing IOP [10,12,26]. PEXG, the most common secondary open-angle glaucoma worldwide, is known for its greater surgical complexity. To the best of our knowledge, few studies have compared PFMS outcomes in patients with PEXG and POAG, often with imbalanced subgroups, limiting the strength of their conclusions. According to the 2022 expert consensus, PEXG is an indication for the – currently off-label – implantation of PFMS [27]. Given the increased surgical challenges of PEXG compared to POAG, robust comparative data are essential. This study bridges that gap and provides real-world clinical insights.

PFMS is suitable for patients needing a safer alternative to trabeculectomy when target IOP is in the middle teens. We analysed 25 eyes per group, ensuring equal representation of POAG and PEXG. Preoperative IOP in the POAG group was 25.12 mmHg and in the PEXG group 25.64 mmHg (p = 0.98), slightly higher than in previous studies on PFMS [20,22,28]. This reflects our study’s inclusion of all consecutive PEXG patients who underwent PFMS surgery with at least one year of follow-up, each matched with a corresponding POAG patient from our database. Because PEXG is often associated with higher preoperative IOP, POAG eyes with similarly elevated IOP were selected to ensure a well-balanced matched-pair analysis and maintain comparability between groups (Table 1). During the initial months following surgery, no significant differences were observed between the two groups. However, after one year, eyes with POAG had lower IOP compared to eyes with PEXG (12.64 mmHg vs. 15.04 mmHg; p = 0.043) and required fewer IOP-lowering medications (0.52 vs. 1.30; p = 0.031). Consequently, significant differences were found in the qualified success criteria for A and B (p = 0.039 and p = 0.024) for POAG. This difference may reflect the more aggressive nature of PEXG and its distinct pathophysiology. Progressive deposition of pseudoexfoliative material and increased postoperative fibrosis may impair aqueous outflow through the PreserFlo MicroShunt or promote bleb failure, thereby reducing long-term surgical efficacy. These potential mechanisms are discussed in more detail below and may also explain the higher rate of postoperative interventions observed in the PEXG group.

Several studies report that PFMS has comparable IOP-lowering effects in both POAG and PEXG. Nobl et al. demonstrated effective IOP-reduction in both groups with baseline values of 21.5 ± 5.8 mmHg (PEXG) and 18.2 ± 4.5 mmHg (POAG) at baseline to 12.8 ± 3.0 mmHg and 12.9 ± 4.2 mmHg, respectively [20]. Storp et al., however, reported a greater reduction in IOP for PEXG (from 24.0 mmHg to 13.0 mmHg for PEXG and from 21.0 mmHg to 14.0 mmHg for POAG) [28]. Fea et al. reported an IOP-lowering effect from 25.0 ± 6.7 mmHg to 14.3 ± 3.6 mmHg for POAG and from 25.0 ± 5.9 mmHg to 13.5 ± 2.4 mmHg for PEXG after 12 months [22]. In contrast, our findings indicate a more pronounced IOP reduction in POAG, aligning with results from studies evaluating other glaucoma procedures [19,29,30]. Discrepancies between our findings and those of the previously mentioned studies may be attributed to differences in study design. Our study ensured equal representation and matched-pair analysis, whereas others did not include a group of POAG patients for comparison [31] or had imbalanced subgroups [22,28], potentially leading to discrepancies in baseline characteristics. In the absence of appropriate matching, confounding factors such as variations in disease severity, preoperative IOP, lens status and history of glaucoma surgery could have influenced the reported outcomes [20,22,28,31]. By carefully pairing patients based on preoperative parameters and ensuring comparability, our study allows for a more reliable comparison of PFMS efficacy between POAG and PEXG.

Some studies suggest poorer PFMS outcomes in PEXG compared to POAG. Rabiolo et al. identified PEXG and pigmentary glaucoma as risk factors for failure (HR = 1.641, p = 0.004). Further risk factors were primary angle closure glaucoma (HR = 1.611, p < 0.001) and previous non-glaucomatous ocular surgeries (HR = 2.301, p = 0.002) [32]. The postoperative outcomes of PFMS in Asian patients with PEXG demonstrated an approximate 50% success rate at both 24 and 48 weeks, with a reoperation rate of approximately 31%. Both glaucoma subtype and ethnicity may have contributed to poorer outcomes [31].

In PEXG, extracellular material is known to accumulate on various ocular structures, including the anterior lens capsule, pupil margin, lens zonules, and the anterior chamber angle. Given this widespread deposition, it is reasonable to consider that similar material could also accumulate at the entry point of the PFMS into the anterior chamber or even within the device itself. Such obstruction could compromise aqueous outflow obstruction may potentially compromise aqueous outflow and contribute to less favourable surgical outcomes in PEXG patients compared to those with POAG. This may, at least in part, explain the differences observed in postoperative IOP reduction and the increased need for additional interventions in PEXG eyes [33,34].

One of the key advantages of PFMS over trabeculectomy is its superior safety profile, particularly in minimizing the risk of postoperative hypotony, uveal effusion, and suprachoroidal haemorrhages, which can lead to vision loss. However, our study revealed a significantly higher incidence of complications in eyes with PEXG compared to POAG (48.0% vs. 20.0%, p = 0.038). Notably, these included transient complications such as hyphema and corneal erosions in both groups, all of which were self-resolving and did not require further intervention. Given that PFMS is a penetrating glaucoma surgery with the tube positioned in the anterior chamber, hyphema occurrence is expected. Moreover, the relatively high prevalence of patients on blood-thinning therapy in both groups (32.0% vs. 28.0%) may have contributed to this complication. Similar rates of hyphema have been described in the literature [22,28].

In our study, two eyes in the POAG group and one in the PEXG group developed self-limiting hypotony accompanied by choroidal detachment. One eye in the POAG group required an injection of viscoelastic to manage persistent hypotony. Importantly, none of these eyes experienced “kissing choroids” or any vision-threatening complications related to hypotony. Nobl et al. reported a hypotony rate for PEXG (IOP < 6 mmHg at any time) of 40.0% in their study with a choroidal detachment rate of 30.0% and a shallow anterior chamber rate of 15.0% [20]. Fea et al. described a lower rate of choroidal detachments with 4.8% and choroidal haemorrhage of 1% [22]. Storp et al. reported postoperative adverse events in 71% of eyes with PEXG [28], while Wakuda et al. state a hypotony rate of 17% and development of choroidal detachment in 3.5% in eyes with PEXG [31]. Our findings align with these studies, placing our complication rates within the range described in the literature.

In our study, postoperative procedures were more frequently performed in eyes with PEXG (32.0%) compared to those with POAG (4.0%) (p = 0.023). These procedures were predominantly revisions (one eye with POAG (4.0%) vs. five eyes with PEXG (20.0%)), with needlings and additional glaucoma surgeries required exclusively in the PEXG group (8.0% and 4.0%, respectively). This contrasts with findings from other studies which report on lower revision rates, while needling rates were higher. Fea et al. reported on 13.5% of patients after PFMS had a revision that needed an opening of the conjunctiva and 18.3% that received a needling, without differentiating between the glaucoma subtypes [22]. Nobl et al. described that 34.6% of all patients with POAG needed a needling, while it was 25.0% of the patients with PEXG [20]. A surgical revision was necessary in 7.69% (POAG) and 10.0% (PEXG). Storp et al. report revision or further glaucoma surgery in 23.0% of POAG eyes and 39.0% of PEXG eyes [28]. Wakuda et al. also report similar numbers with a reoperation rate of 31%, with 24.1% of cases being revisions [31]. The frequency of needlings for eyes with POAG in further studies ranges between 5% and 19% in the first 12 months after PFMS surgery [6,35]. In our experience, surgical revision was more efficient than needling, especially when encapsulation was present following PFMS implantation. As a result, our data may reflect a higher number of revisions than needlings, which contrasts with other studies that report a greater frequency of needling as a secondary procedure.

Three eyes (12.0%) in the PEXG group experienced failure, requiring additional glaucoma surgery. Two eyes received a PAUL glaucoma implant and one eye an eyePlate implant. There was no significant difference in the history of prior glaucoma surgery between the two groups, and none of the eyes had undergone vitrectomy. These findings are consistent with those from other studies. Storp et al. described that 7.0% needed another glaucoma surgery during the first 12 months [28]. Fea et al. reported on lower rates with 3.8% that had a trabeculectomy or cyclophotocoagulation [22]. Both studies did not differentiate between glaucoma subtypes. If larger cohorts are considered for POAG, the rates vary between 5% and 15% [11,23,35]. Our results suggest that eyes with PEXG may be more likely to require additional postoperative surgical intervention than eyes with POAG, indicating poorer surgical outcomes in this cohort.

This study demonstrates that PFMS is an effective intervention for lowering IOP in both POAG and PEXG patients. However, POAG patients experienced greater IOP reduction and needed less IOP-lowering medications. Although the difference in IOP between the two groups was statistically significant at 12 months, the actual numerical difference was only 2.4 mmHg. Importantly, both groups achieved IOP reductions to the mid to low teens, supporting PFMS as a viable and effective treatment option for both POAG and PEXG patients.

There are several limitations of this study. Firstly, its retrospective design inherently restricts the analysis, and we were only able to report one-year outcomes, thus long-term outcomes will have to be re-evaluated over time. Moreover, five patients after PFMS had to be excluded from this study due to missing follow-up, one patient died during the postoperative course. This can introduce a certain selection bias, as patients without postoperative complications are more likely to be lost to follow-up and continue care with their local ophthalmologist. Furthermore, some variables of interest, such as endothelial cell counts, were not available since they were not routinely performed for all patients. Due to the retrospective design of the study, reliable and standardized data on postoperative bleb morphology were not consistently available and therefore could not be analysed. Moreover, the time since diagnosis was significantly shorter in the PEXG group, indicating a longer duration of topical IOP-lowering medications in eyes with POAG. Consequently, chronic conjunctival inflammatory changes may have been more pronounced in the POAG group, potentially confounding the results. Lastly, the use of MMC has a relevant influence on the success of the PFMS procedure, but there is currently no consensus on dosage and duration of exposure. A standardized MMC concentration of 0.2 mg/ml was used in this study. Therefore, no statement can be made as to whether a different concentration would be more effective for PEXG.

## Conclusion

In conclusion, this retrospective, comparative study demonstrates that PFMS effectively lowers IOP and reduces the need for IOP-lowering medications in both POAG and PEXG patients. The procedure maintains a strong safety profile, with no vision-threatening complications observed in either group. However, the IOP-lowering effect was less pronounced in PEXG eyes compared to POAG eyes, and postoperative interventions were required more frequently in the PEXG group, likely due to a higher incidence of bleb scarring. These findings suggest that when choosing a surgical approach, clinicians should consider the increased risk of postoperative complications and IOP fluctuations in PEXG patients. Tailoring postoperative management strategies to address these challenges may further optimize outcomes for this patient group.
